# Adrenal gland size in obstructive sleep apnea: Morphological assessment of hypothalamic pituitary adrenal axis activity

**DOI:** 10.1371/journal.pone.0222592

**Published:** 2019-09-20

**Authors:** Takuma Minami, Ryo Tachikawa, Takeshi Matsumoto, Kimihiko Murase, Kiminobu Tanizawa, Morito Inouchi, Tomohiro Handa, Toru Oga, Toyohiro Hirai, Kazuo Chin

**Affiliations:** 1 Department of Respiratory Medicine, Graduate School of Medicine, Kyoto University, Kyoto, Japan; 2 Department of Respiratory Care and Sleep Control Medicine, Graduate School of Medicine, Kyoto University, Kyoto, Japan; 3 Department of Respiratory Medicine, Kawasaki Medical School, Kurashiki, Japan; University of Rome Tor Vergata, ITALY

## Abstract

**Objectives:**

The association of obstructive sleep apnea (OSA) with hypothalamic pituitary adrenal (HPA) axis activation has not been fully understood from results of previous studies using hormonal assessments. We aimed to investigate the relationship between adrenal size, a potential marker reflecting HPA axis activity, and sleep parameters related to OSA.

**Methods:**

We retrospectively reviewed data on 284 consecutive adult patients aged 20 to 80 y who had undergone polysomnography and abdominal computed tomography (CT). OSA was defined as none/mild (apnea-hypopnea index [AHI] <15, n = 75), moderate (AHI 15 to 30, n = 80), and severe OSA (AHI ≥30, n = 129). Widths of adrenal body and limbs were measured by abdominal CT.

**Results:**

Adrenal size was greater in participants with severe OSA than in those with none/mild or moderate OSA (adrenal body width: 6.03 mm, none/mild OSA; 6.09 mm, moderate OSA; 6.78 mm, severe OSA; p <0.001; adrenal limb width: 3.75 mm, none/mild OSA; 3.95 mm, moderate OSA; 4.26 mm, severe OSA, p <0.001). Multivariate regression analysis showed that not the 3% oxygen desaturation index and time of SpO_2_ <90% but a higher arousal index was the only determinant factor for increased adrenal limb width (β = 0.27, p <0.001) after adjusting for other variables that could affect adrenal size. Neither the arousal index nor hypoxic parameters were associated with adrenal body width.

**Conclusions:**

Results indicated that adrenal glands may enlarge in response to longstanding sleep fragmentation, suggesting the involvement of OSA in HPA axis augmentation.

## Introduction

Obstructive sleep apnea (OSA) is a prevalent sleep disordered breathing condition characterized by repetitive partial or complete upper airway obstruction during sleep. Intermittent hypoxia and sleep fragmentation are the major pathophysiological consequences of OSA that induce sympathetic nervous hyperactivity, oxidative stress, and systemic inflammation, leading to metabolic and cardiovascular comorbidities [1,2].

OSA-induced hypoxia and sleep fragmentation are considered to be stressors that may activate human stress systems consisting of the sympathetic nervous system and hypothalamic pituitary adrenal (HPA) axis. The activation of stress systems in OSA has been well demonstrated in the sympathetic nervous system and may contribute to the development of comorbidities [3]. On the other hand, the results of previous studies that investigated the effects of OSA on the HPA axis were inconsistent, presumably due to the circadian rhythm and fluctuating nature of cortisol secretion [4]. Although studies demonstrating that elevated cortisol levels in OSA patients might support a temporal association between OSA and HPA axis activation [5,6], those findings do not necessarily reflect accumulated effects of OSA as a stressor. In addition, which sleep index in OSA that serves as a strong physiological stressor to activate the HPA axis has not been identified.

Adrenal glands increase in size under stressful conditions [7]. Their size can be measured by abdominal computed tomography (CT) [8] or magnetic resonance imaging [9], and their enlargement was reported in several clinical stressful settings such as malignancy, depression, infectious diseases, and smoking [10–15]. In addition, patients with metabolic and adrenocortical endocrine dysfunctions have enlarged adrenal glands [16–19]. Thus, adrenal enlargement could be regarded as a manifestation of HPA axis hyperactivity.

We hypothesized that adrenal size could be a potential stress indicator that reflects longstanding physiological stress due to OSA and that adrenal glands would be enlarged in OSA patients due to chronic sleep disordered breathing. The present study aimed to investigate the associations between adrenal size assessed by CT and sleep parameters in patients with OSA.

## Methods

### Study participants

**Fig 1:**
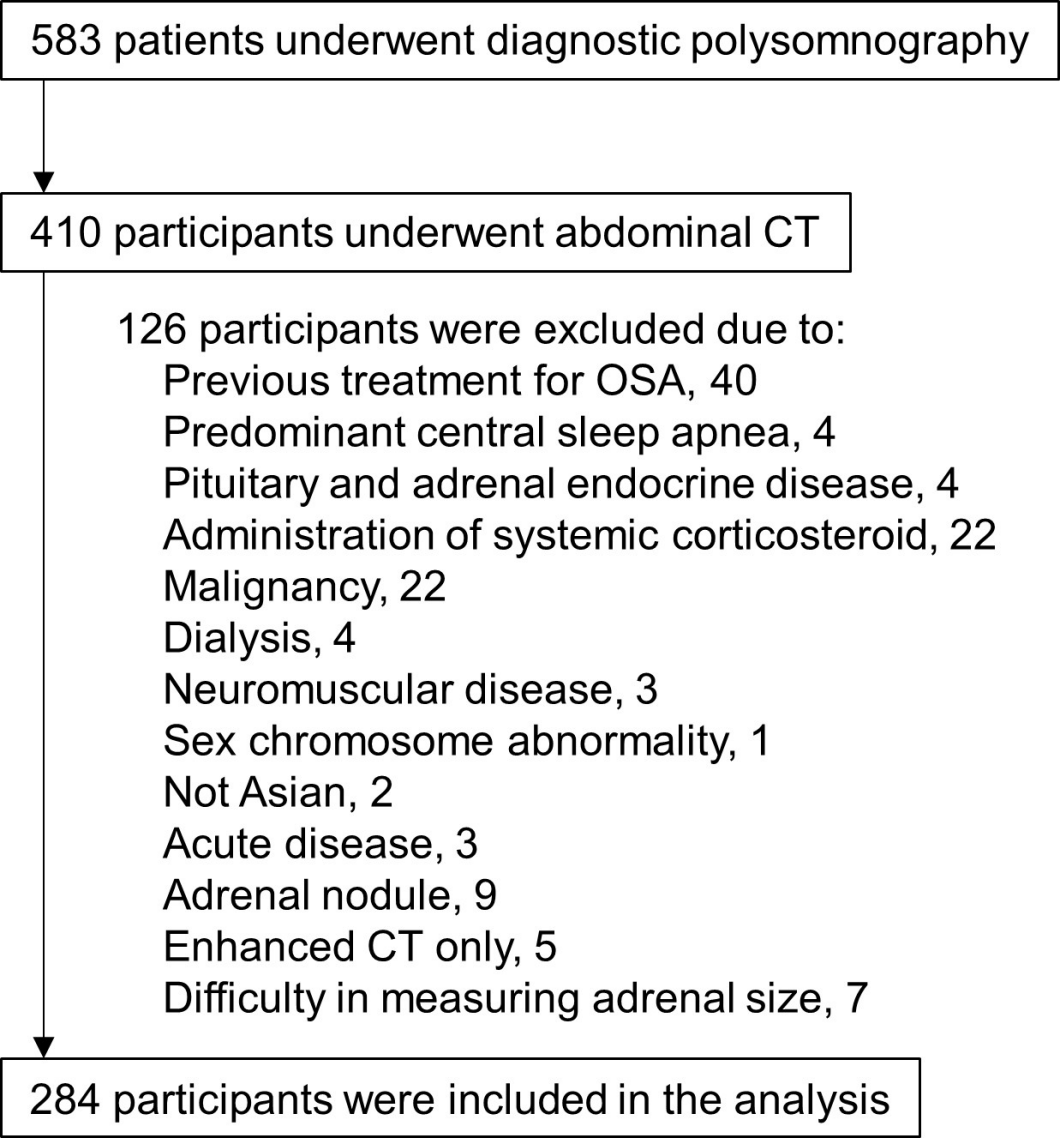
Flowchart of patient enrollment in the present study. CT: computed tomography, OSA: obstructive sleep apnea.

We retrospectively reviewed adult patients aged 20 to 80 y who were consecutively admitted to Kyoto University Hospital to undergo diagnostic polysomnography from November 2008 to December 2010. We recommended patients with suspicion of metabolic syndrome to undergo abdominal CT to evaluate the visceral fat area because the prevalence of metabolic syndrome in moderate to severe OSA patients is about 50% [20,21], and the Japanese criteria for metabolic syndrome includes a visceral fat area ≥100 cm^2^ [22]. Of the 583 patients who underwent diagnostic polysomnography, we selected the 410 patients who also underwent abdominal CT before treatment for OSA as possible study participants (410/583: 70.3%) (Fig 1). The 410 participants who had abdominal CT were older tendency and more obese than the 173 participants who did not have abdominal CT (age: 60.0 [50.0–69.0] y vs. 58.0 [41.0–69.5] y, p = 0.057; body mass index: 25.6 [23.1–29.1] kg/m^2^ vs. 24.7 [21.9–28.3] kg/m^2^, p = 0.014). The proportion of men who underwent abdominal CT was not different from those who did not (men: 71.0% vs. 69.4%, p = 0.70). From the 410 participants who had undergone abdominal CT, we excluded 126 participants for the following reasons: previous treatment for OSA, predominant central sleep apnea, pituitary or adrenal endocrine diseases, administration of systemic corticosteroid, malignancy or treatment history of malignancy within 5 years, dialysis, neuromuscular diseases, sex chromosome abnormality, not Asian, abdominal CT in the acute disease phase, adrenal nodule, only underwent enhanced CT, and undetectable adrenal glands. Finally, 284 participants were included in the analysis (Fig 1). Kyoto University Graduate School and Faculty of Medicine, Ethics Committee approved the present study. Because the present study was a retrospective analysis, and we did not obtain consent of each participant. We disclosed the research information in web page of our institution and give each participant a chance to opt-out instead.

### Measurement of adrenal glands and visceral fat area

**Fig 2:**
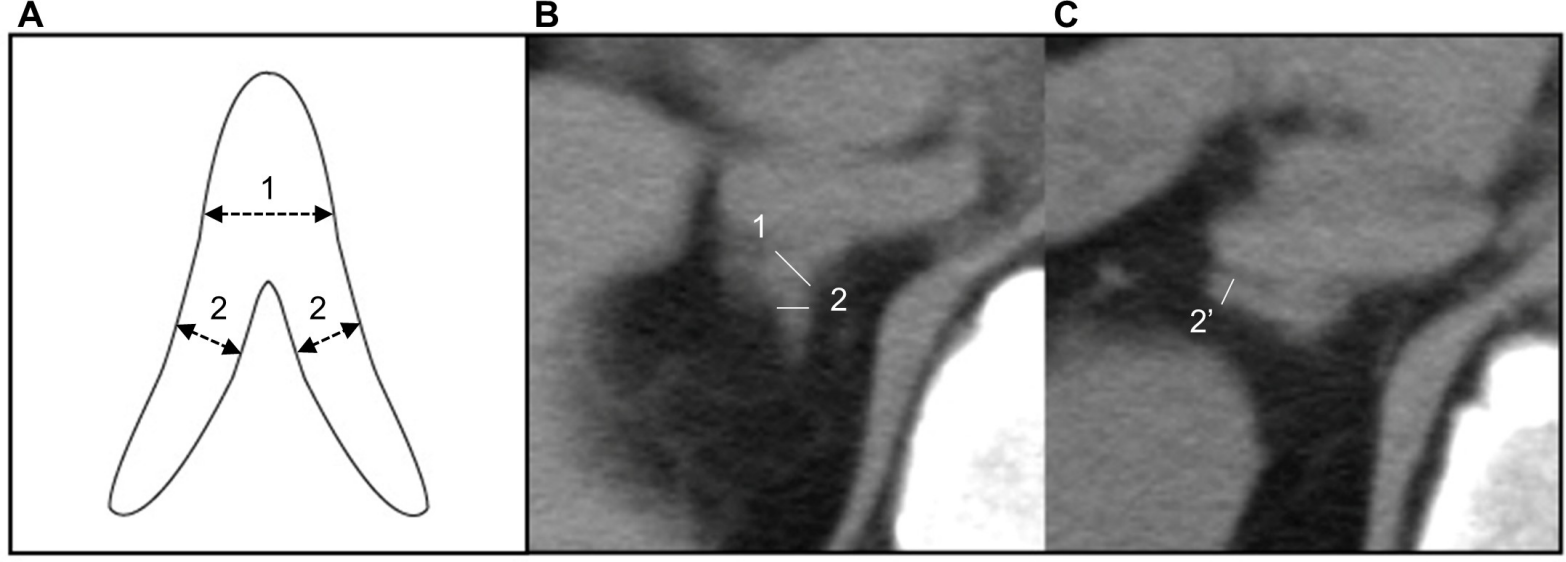
Measurements of the adrenal gland. (A) Scheme of adrenal measurement: 1 = width of body; 2 = width of limb. (B) White line shows the width of the right adrenal body [1] and right medial limb [2]. (C) White line shows the width of the right lateral limb [2’]. Different slices were selected to measure the width of each part at the widest point.

All abdominal CT studies to measure abdominal visceral fat areas were conducted before the initiation of OSA treatment. Participants were assessed with unenhanced abdominal CT using a 64-slice multidetector CT scanner (Aquilion 64; Toshiba, Tokyo, Japan). Adrenal size was measured in accordance with previous reports [8]. CT scans with a 7-mm slice thickness with conditions of window width of 400 Hounsfield units and window level of 40 Hounsfield units were examined by one analyzer. The analyzer was blinded to OSA severity at the time of the radiologic interpretation and measured three parts of the bilateral adrenal glands: medial limb, lateral limb, and adrenal body (Fig 2). The width of each part was measured perpendicularly against the long axis at the widest point. We defined “mean body width” as the mean of bilateral body widths and “mean limb width” as the mean of measurable bilateral limb widths. An adrenal nodule was defined as a round adrenal mass with a major axis of 1 cm or larger. We could not detect an adrenal gland in 7 participants due to the paucity of retroperitoneal fat or probable hypoplasia. Of 284 eligible participants, we could measure the body width of each adrenal gland. We could not identify the lateral limb in 52 participants and the medial limb in 4 participants for the right adrenal gland and the lateral limb in 8 participants and the medial limb in 9 participants for the left adrenal gland. We could measure at least two or three limbs in all participants. The intra-observer percentage differences in mean body and limb widths from 30 random participants were 6.4% and 6.0%, respectively.

We also measured the visceral fat area from a CT slice at the umbilicus level as described previously [23].

### Polysomnography

All participants underwent overnight polysomnography (SomnoStar pro, Cardinal Health, Dublin, OH, USA or Alice 4, Philips Respironics, Inc., Murrysville, PA, USA) in Kyoto University Hospital. Apnea was defined as a decrease in airflow by ≥90% of baseline for more than 10 s. Hypopnea was defined as a decrease in airflow by ≥50% of baseline for more than 10 s with ≥3% desaturation and/or arousal from sleep. Apnea hypopnea index (AHI) was calculated according to episodes of apnea and hypopnea per h over total sleep time [24]. OSA severity was defined as none/mild (AHI <15), moderate (AHI 15 to 30), and severe (AHI ≥30). 3% oxygen desaturation index (ODI) was calculated according to episodes of ≥3% desaturation per h over total sleep time. Arousal during sleep stages was defined as an abrupt shift in the electroencephalogram such as alpha lasting for >3 s. The arousal index was calculated according to episodes of arousals per h over total sleep time.

### Additional data collected

Anthropometric parameters and questionnaires (Japanese version of the Epworth Sleepiness Scale and the Hospital Anxiety and Depression Scale) were measured on the day of admission for polysomnography. The Japanese version of the Epworth Sleepiness Scale was validated in Japanese [25], and a total score ≥11 indicates excessive daytime sleepiness. The Hospital Anxiety and Depression Scale (HADS) is a 14-item self-report questionnaire comprising 7 anxiety items and 7 depression items from which subscale scores for anxiety and depression are separately calculated [26]. Subscores of 8–10 indicate possible anxiety or depression. In the present study, we defined the presence of anxiety or depression as a score of ≥8 for the related subscore.

Because this study was conducted retrospectively, the following data were missing: waist circumference in 9 patients; smoking status in 3 patients; HADS in 30 patients; and the Japanese version of the Epworth Sleepiness Scale in 29 patients.

### Definition of comorbidities

We defined obesity as body mass index >25 kg/m^2^ [27]; hypertension as the usage of antihypertensive drugs or systolic blood pressure ≥140 mmHg and/or diastolic blood pressure ≥90 mmHg; dyslipidemia as the use of lipid lowering drugs or low density lipoprotein cholesterol ≥140 mg/dl, high density lipoprotein cholesterol <40 mg/dl or triglycerides ≥150 mg/dl; and diabetes mellitus as the use of hypoglycemic drugs including insulin or HbA1c ≥6.5% with fasting plasma glucose ≥126 mg/dl. We also defined metabolic syndrome in reference to Japanese recommended criteria as the presence of visceral obesity (visceral fat area of abdominal CT at umbilicus slice ≥100 cm^2^), which is an essential component, and of at least two of the following metabolic abnormalities: systolic blood pressure ≥130 mmHg and/or diastolic blood pressure ≥85 mmHg and/or hypertension; fasting plasma glucose ≥110 mg/dL and/or diabetes; triglycerides ≥150 mg/dL and/or high-density lipoprotein <40 mg/dL and/or the use of drugs for such conditions (e.g. fibrates) [22].

### Statistical analysis

Results are shown as median (IQR) or the number of participants (%) according to OSA severity. We used the Kruskal-Wallis test to compare continuous variables as appropriate and the Chi-square test to compare categorical variables. We used the Steel-Dwass test to perform post hoc pairwise comparisons among groups. Pearson correlation analysis and Spearman rank test were used to determine the correlation between adrenal size and other variables. Next, we conducted multivariate linear regression analysis to evaluate the relationship between adrenal size and sleep parameters. In model 1, the AHI was selected as a representative sleep parameter of OSA severity. In models 2 and 3, we further investigated the impacts of hallmarks of OSA, which are intermittent hypoxia and sleep fragmentation, on adrenal size. In model 2, the 3% ODI (index of intermittent hypoxia) and time of SpO2 <90% (index of sustained hypoxia) were included instead of the AHI. In model 3, we only included the arousal index (index of sleep fragmentation). Other covariates included age, sex, and factors known to be correlated with adrenal size (obesity, smoking, and depression) [11,15,28]. From the results of the univariate analysis, we decided to choose one variable to be included in the multivariate regression out of obesity-related parameters such as body mass index, waist circumference, and visceral fat area. P value <0.05 was considered significant in all analyses. We performed all statistical analyses by JMP 11.2.0 [Statistical Analysis System (SAS) Institute, Inc., Cary, NC, USA].

## Results

### Participants’ characteristics

Characteristics of the participants are shown in Table 1. Participants with severe OSA were older, more obese, and included a higher proportion of men, a greater percentage of those with hypertension, and had more pack years of smoking than those with none/mild or moderate OSA. There were no significant differences in sleepiness and depression scores among groups whereas the anxiety score was higher in participants with none/mild OSA than in those with severe OSA.

**Table 1:** Characteristics of study participants according to OSA severity.

	None/mild OSA	Moderate OSA	Severe OSA	P value
	n = 75	n = 80	n = 129	
**Clinical background**				
Age (years)	54.0 (41.0–66.0)	59.5 (46.3–69.0)	60.0 (52.0–68.0)*	<0.001
Men, n	44 (59%)	59 (74%)	110 (85%)	<0.001
Body mass index (kg/m^2^)	24.9 (21.8–27.6)	25.2 (22.8–26.9)	27.1 (24.4–30.6)*^†^	<0.001
Waist circumference (cm)	89.0 (82.5–97.0)	91.0 (85.8–97.3)	96.0 (89.6–105.8)*^†^	<0.001
Visceral fat area (cm^2^)	92.0 (50.5–124.5)	100.1 (64.4–129.2)	132.0 (87.9–172.2)*^†^	<0.001
Smoking				
Current/Past/Never, n	13/21/40	6/37/36	23/59/46	0.012
Pack-years	0.0 (0.0–20.0)	2.0 (0.0–30.0)	10.0 (0.0–33.5)	0.013
**Comorbidity**				
Obesity, n	37 (49%)	42 (53%)	89 (69%)	0.008
MetS, n	13 (17%)	26 (33%)	58 (45%)	<0.001
Hypertension, n	29 (39%)	47 (59%)	87 (67%)	<0.001
Dyslipidemia, n	48 (64%)	58 (73%)	95 (74%)	0.33
Diabetes mellitus, n	11 (15%)	15 (19%)	35 (27%)	0.085
**Questionnaire**				
JESS	10.0 (7.0–14.0)	10.5 (6.0–13.0)	8.5 (6.0–13.0)	0.32
EDS, n	33 (46%)	34 (50%)	46 (40%)	0.36
HADS-A	6.0 (3.0–9.0)	5.5 (3.0–8.0)	5.0 (2.0–7.0)*	0.021
Anxiety, n	28 (49%)	20 (29%)	26 (22%)	0.040
HADS-D	6.0 (3.0–9.0)	5.0 (3.0–8.0)	3.0 (2.0–5.0)	0.29
Depression, n	25 (36%)	20 (29%)	33 (28%)	0.57
**Sleep parameters**				
AHI (events/h)	8.4 (4.7–11.6)	22.6 (20.6–26.9)*	46.2 (38.0–57.2)*^†^	<0.001
3% ODI (events/h)	5.8 (3.2–9.4)	19.0 (16.3–22.6)*	43.8 (34.1–56.0)*^†^	<0.001
Time of SpO_2_ <90% (%)	0.2 (0.0–1.1)	1.9 (0.6–4.8)*	13.8 (4.5–33.5)*^†^	<0.001
Arousal index (events/h)	17.0 (13.0–23.8)	24.4 (16.9–33.5)*	40.1 (28.8–50.9)*^†^	<0.001

Data are expressed as median (IQR) or number (%).

Kruskal-Wallis test was used to compare the medians among groups.

*P <0.05 vs. None/Mild

^†^P <0.05 vs. Moderate

Definition of abbreviations: MetS = metabolic syndrome; JESS = Japanese version of the Epworth Sleepiness Scale; EDS = excessive daytime sleepiness; HADS-A = Hospital Anxiety and Depression Scale for anxiety; HADS-D = Hospital Anxiety and Depression Scale for depression; AHI = apnea-hypopnea index; ODI = oxygen desaturation index.

### Adrenal measurements and their correlations with variables

**Fig 3:**
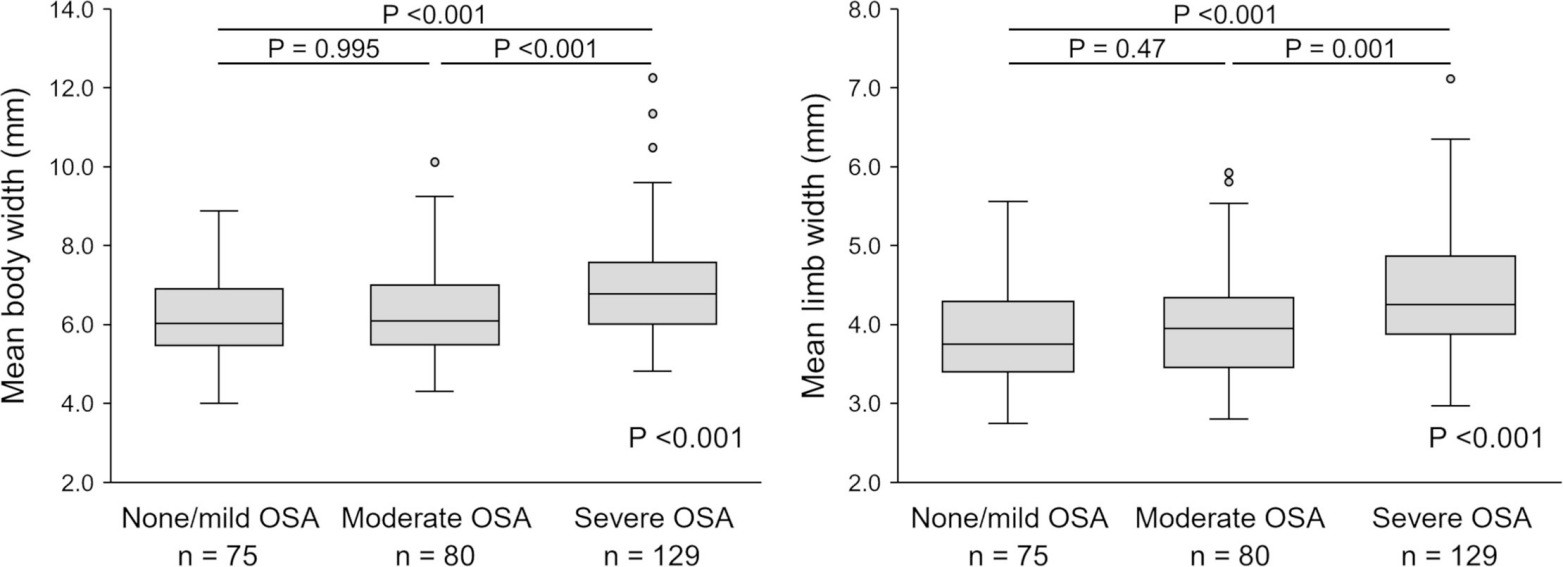
Mean body and limb width according to OSA severity. The box in box plots shows the IQR and the line within the box shows the median. The upper whisker shows the 75^th^ percentile plus 1.5 x IQR, and the lower whisker shows the 25^th^ percentile minus 1.5 x IQR. Dots indicate the outliers. Analyses were conducted by the Kruskal-Wallis test and Steel-Dwass test for post hoc pair wise comparisons.

**Fig 4:**
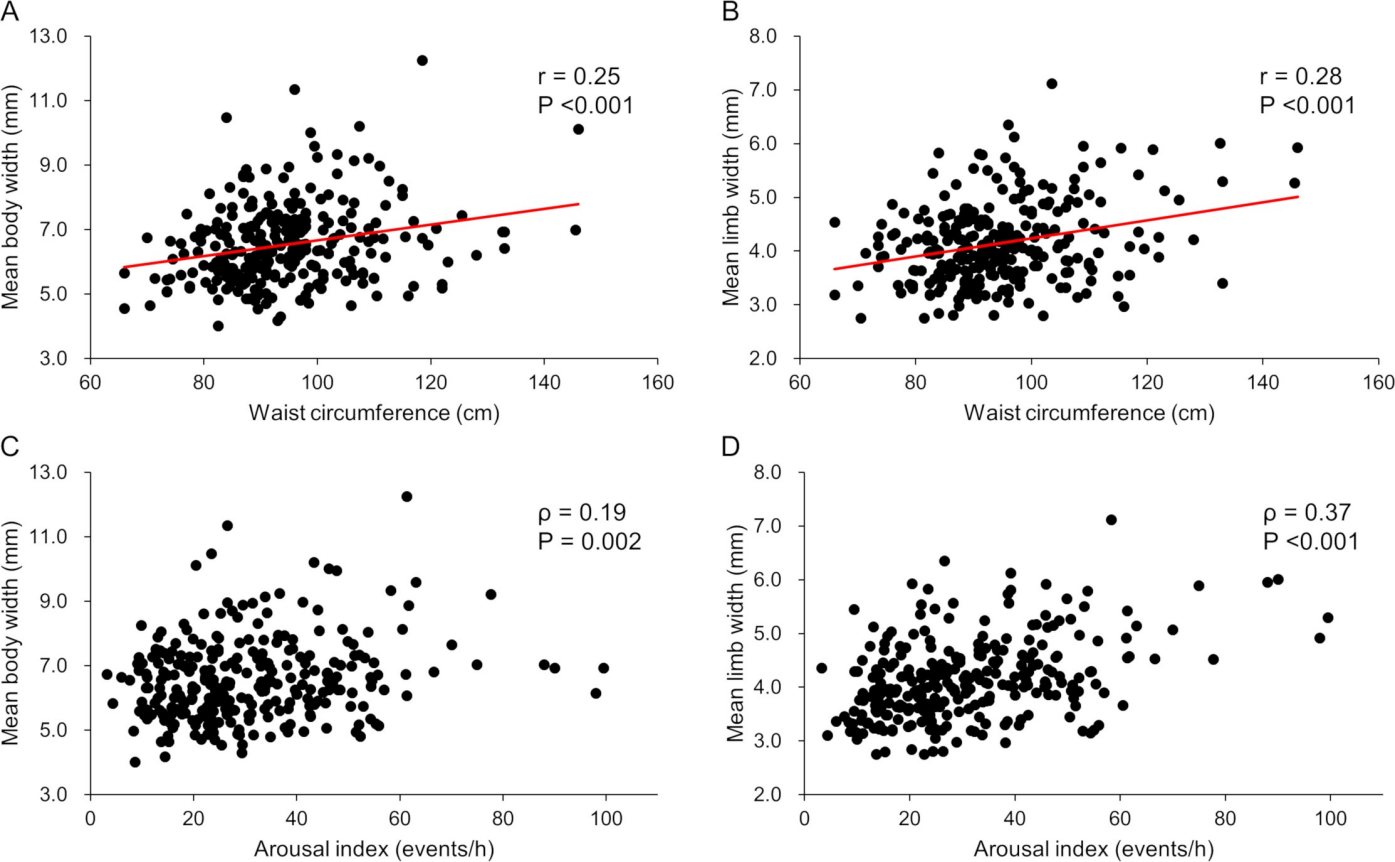
Correlations between mean adrenal limb width or mean adrenal body width and representative sleep and obesity related parameters. (A) Correlation between waist circumference and mean body width. (B) Correlation between waist circumference and mean limb width. (C) Correlation between arousal index and mean body width. (D) Correlation between arousal index and mean limb width. Correlations between adrenal measurements and variables are shown by Pearson correlation analysis (r) or Spearman rank test (ρ).

Mean adrenal body and limb widths in severe OSA participants were greater than in those with none/mild and moderate OSA (body width: 6.03 [5.48–6.91] mm, none/mild OSA; 6.09 [5.49–6.99] mm, moderate OSA; and 6.78 [6.01–7.58] mm in severe OSA; p <0.001; limb width: 3.75 [3.40–4.29] mm, none/mild OSA; 3.95 [3.45–4.34] mm, moderate OSA; and 4.26 [3.88–4.87] mm, severe OSA; p <0.001) (Fig 3). There were 21 and 54 patients in the none and mild OSA groups, respectively. Mean body width and mean limb width (none vs. mild OSA) were: mean body width 5.64 (5.20–6.53) vs. 6.31 (5.53–6.99), p = 0.056; 3.70 (3.27–4.18) vs. 3.87 (3.48–4.31), p = 0.28. Although the none OSA group was small and we analyzed as none/mild OSA, the trends of an increase in adrenal size according to OSA severity was preserved in the none and mild OSA groups. In the univariate analysis, positive correlations were noted for both body and limb width with the following variables: age, male sex, current smoker, body mass index, waist circumference, visceral fat area, AHI, time of SpO_2_ <90%, 3% ODI, and the arousal index (Table 2 and Fig 4). Correlations with these variables were relatively stronger for limb width compared to body width. Details of the correlations among adrenal size and obesity-related parameters were as follows: body mass index (mean body width: r = 0.14, p = 0.017; mean limb width: r = 0.21, p <0.001), waist circumference (mean body width: r = 0.25, p <0.001; mean limb width: r = 0.28, p <0.001), and visceral fat area (mean body width: r = 0.12, p = 0.036; mean limb width: r = 0.32, p <0.001) (Table 2 and Fig 4).

**Table 2:** Correlations between mean adrenal limb width or mean adrenal body width and variables.

**Variables**	**Mean body width**		**Mean limb width**	
	**r**	**P value**	**r**	**P value**
Age (years)	0.13	0.025	0.21	<0.001
Body mass index (kg/m^2^)	0.14	0.017	0.21	<0.001
Visceral fat area (cm^2^)	0.12	0.036	0.32	<0.001
	**ρ**	**P value**	**ρ**	**P value**
Sex (men)	0.13	0.03	0.19	0.001
Current smoker	0.07	0.22	0.07	0.28
Obesity (+)	0.06	0.29	0.15	0.014
MetS (+)	0.08	0.17	0.13	0.031
Anxiety (+)	-0.08	0.18	-0.01	0.85
Depression (+)	0.02	0.75	0.03	0.61
AHI (events/h)	0.22	<0.001	0.29	<0.001
3% ODI (events/h)	0.19	0.001	0.27	<0.001
Time of SpO_2_ <90% (%)	0.19	0.001	0.26	<0.001

Correlations between adrenal measurements and variables are shown by Pearson correlation analysis (r) or Spearman rank test (ρ). Definition of abbreviations: MetS = metabolic syndrome; AHI = apnea-hypopnea index; ODI = oxygen desaturation index.

Obesity (+) = a participant who has body mass index >25 kg/m^2^; MetS (+) = a participant who has metabolic syndrome; anxiety (+) = a participant who has Hospital Anxiety and Depression Scale for anxiety >8; depression (+) = a participant who has Hospital Anxiety and Depression Scale for depression >8.

### Multivariate regression

We included waist circumference as a variable in the multivariate regression from the obesity-related parameters because it had a moderate and almost the same degree of correlations with both body and limb width in the univariate analysis. Models of multivariate regression analysis are shown in Table 3. After adjusting for the confounders, the AHI was associated with not adrenal body width but adrenal limb width (β = 0.14, p = 0.046). While the 3% ODI and time of SpO_2_ <90% were not associated with either adrenal body width or limb width, the arousal index was an independent predictor of an increased adrenal limb width (β = 0.27, p <0.001) but was not associated with body width (β = 0.04, p = 0.53). Except for sleep parameters, age and waist circumference were consistently associated with increased size of both the adrenal body and limb in all models. The correlation of the arousal index with adrenal limb width was almost the same (β = 0.29, p <0.001) when the visceral fat area was used in models of limb width despite the values for waist circumference (S1 Table).

**Table 3:** Multivariate regression analysis for mean of bodies and limbs. n = 244.

**Mean body width**						
**Variables**	**Model 1**		**Model 2**		**Model 3**	
	**ß**	**P value**	**ß**	**P value**	**ß**	**P value**
Age (years)	0.20	0.002	0.21	0.001	0.19	0.005
Sex (men)	0.14	0.026	0.15	0.017	0.13	0.041
Waist circumference (cm)	0.39	<0.001	0.40	<0.001	0.37	<0.001
Current smoker	0.06	0.34	0.07	0.30	0.05	0.41
Obesity (+)	-0.07	0.39	-0.06	0.45	-0.07	0.36
MetS (+)	-0.06	0.40	-0.06	0.37	-0.06	0.43
Depression (+)	0.03	0.62	0.03	0.63	0.04	0.55
AHI (events/h)	-0.01	0.91	-	-	-	-
3% ODI (events/h)	-	-	0.03	0.77	-	-
Time of SpO_2_ <90% (%)	-	-	-0.10	0.28	-	-
Arousal index (events/h)	-	-	-	-	0.04	0.53
***R***^***2***^ **(adjusted *R***^***2***^**)**	**0.142 (0.113)**		**0.147 (0.115)**		**0.143 (0.114)**	
**Mean limb width**						
**Variables**	**Model 1**		**Model 2**		**Model 3**	
	**ß**	**P value**	**ß**	**P value**	**ß**	**P value**
Age (years)	0.25	<0.001	0.25	<0.001	0.19	0.003
Sex (men)	0.16	0.009	0.16	0.009	0.12	0.041
Waist circumference (cm)	0.21	0.014	0.21	0.014	0.19	0.015
Current smoker	0.11	0.065	0.11	0.068	0.09	0.14
Obesity (+)	0.05	0.52	0.04	0.56	0.04	0.60
MetS (+)	-0.02	0.74	-0.02	0.80	0.00	0.99
Depression (+)	0.06	0.32	0.05	0.38	0.08	0.15
AHI (events/h)	0.14	0.046	-	-	-	-
3% ODI (events/h)	-	-	0.04	0.65	-	-
Time of SpO_2_ <90% (%)	-	-	0.11	0.21	-	-
Arousal index (events/h)	-	-	-	-	0.27	<0.001
***R***^***2***^ **(adjusted *R***^***2***^**)**	**0.183 (0.155)**		**0.184 (0.152)**		**0.229 (0.203)**	

Adjusted R^2^ was calculated from R^2^ after adjusting for the number of covariates in models.

Definition of abbreviations: β = standardized partial regression coefficient; MetS = metabolic syndrome; AHI = apnea-hypopnea index; ODI = oxygen desaturation index.

Obesity (+) = a participant who has body mass index >25 kg/m^2^; MetS (+) = a participant who has metabolic syndrome; depression (+) = a participant who has Hospital Anxiety and Depression Scale for depression >8.

## Discussion

This is the first study, to our knowledge, to investigate the relationship between adrenal size and sleep parameters in patients with OSA. We found that the AHI was independently associated with adrenal limb width after adjusting for potential confounders, but not with adrenal body width. Further analyses showed that the arousal index was associated particularly with adrenal limb width. On the other hand, 3% ODI and time of SpO_2_ <90% were not associated with adrenal size. These findings indicate that sleep fragmentation rather than hypoxia may play a major role in inducing morphological changes in adrenal glands.

Previous studies have investigated HPA axis activity in OSA, but results of single-time sampling of cortisol were inconsistent [29–32] partly because cortisol secretion has a distinct circadian rhythm and variations in the cortisol level become prominent before and after waking up [33]. In addition, alterations in sleep duration and sleep architecture inherent to laboratory sleep studies, known as the first-night effect, might well have affected cortisol secretion [34]. Some studies with frequent blood sampling by an indwelling catheter showed that OSA patients had higher cortisol secretion and that withdrawal of continuous positive airway pressure in OSA patients increased nocturnal cortisol secretion, though the procedure was invasive and could be a stressor per se [5,6,35]. Thus, the relationship between OSA and HPA axis activation remains inconclusive with conflicting results [4]. Meanwhile, there is mounting evidence that OSA is robustly associated with sympathetic activation, which is another human stress system. Although secretion of cathecolamines is pulsatile, methods to evaluate autonomic function included not only hormones [36,37] but also physiological indices such as heart rate, blood pressure, and muscle sympathetic nerve activity [38–40]. Since various clinical studies showed that somatic and psychiatric stress increased adrenal size relevant to HPA axis activation [10,12,41], we considered that measuring adrenal size on CT would be a noninvasive method that would not be affected by the fluctuating nature of cortisol secretion and could reflect the cumulative influence of OSA on the HPA axis.

In the current study, we found that the arousal index (i.e., sleep fragmentation) was an independent determinant factor for increased adrenal size in participants with OSA. On the other hand, time of SpO_2_ <90%, and 3% ODI, which are indices of sustained and intermittent hypoxemia, were not associated with adrenal size. Although both intermittent hypoxia and sleep fragmentation were shown to be associated with the activation of the HPA axis in previous studies, the comparison of sleep fragmentation with hypoxia did not show which parameter more strongly activates the HPA axis [42–44]. Our results indicated that long-term sleep fragmentation resulting from OSA could act as a more potent stressor than hypoxia to activate the HPA axis as assessed by adrenal gland enlargement. Keeping in line with this, an association between sleep fragmentation and the sympathetic nervous system, the other stress system, has been demonstrated; chronic fragmented sleep in OSA could increase sympathetic nervous activity independently of hypoxemia during not only sleep but also wakefulness [45,46]. These findings collectively reinforced the importance of incorporating sleep fragmentation into the evaluation of the pathogenic significance of OSA.

Recently, there has been a growing concept that compensatory hyperinsulinemia against insulin resistance accelerates adrenocortical tumor formation through its mitogenic effect [47]. Indeed, 9 of 410 participants had an adrenal nodule in this study (Fig 1). Insulin resistance and metabolic syndrome, which are manifestations of insulin resistance, are common in OSA patients [21]. In the multivariate models, though we used the presence of metabolic syndrome and obesity as possible surrogates of hyperinsulinemia, measurement of insulin would be needed in a future study to carefully investigate the relationship between OSA and adrenal size or the HPA axis.

The result that only adrenal limb width was associated with sleep fragmentation could further support the relationship between OSA and HPA axis activation. The adrenal gland consists of the cortex and medulla. The former, which secretes cortisol, is predominant in the peripheral part (i.e., adrenal limb), while the latter secretes cathecolamines and is predominant in the central part (i.e., adrenal body) [48]. This heterogeneity of adrenal tissue composition and endocrine function may make the adrenal limb more susceptible to HPA axis activation. In fact, adrenal limb width tended to be more enlarged and was associated with endocrine function compared to adrenal bodies [10,17].

Several limitations exist in the present study. First, this was a cross sectional study and did not reveal causality. Although we used multivariate regression analysis to assess the impact of OSA on adrenal size, there might be unknown confounders affecting adrenal size. Second, we did not measure adrenal hormones, and the association of adrenal enlargement with HPA axis activity was not evaluated. However, prior studies showed that adrenal size and HPA axis activation were correlated, supporting our study concept that adrenal enlargement is a marker of chronic HPA axis activation [10,12,17,18]. Third, we did not have enough participants without OSA (AHI <5) to create a none-OSA group separately. Such a reference group might enable us to better understand the relationship between OSA and adrenal size. As mentioned above, participants who did not undergo abdominal CT were younger and leaner than those who had undergone abdominal CT. In Japan, the criteria for metabolic syndrome include waist circumstance ≧85 cm in men or ≥90 cm in women or visceral fat accumulation ≧100 cm^2^ by CT measurement. In addition, inclusion of the latter is recommended if possible [22]. Our CT measurements in patients were performed based on the suspicion of metabolic syndrome and avoiding radiation exposure in those young and not obese patients who would be less likely to have metabolic syndrome. However, some of those patients might have been good candidates for the reference group. Fourth, we did not use volumetric evaluation of adrenal glands, which is more reproducible than linear measurements [28,49]. We could not always capture the outlines of the adrenals and evaluate the volume precisely without enhanced CT because the adrenals often came into contact with other organs such as the liver and vessels. Additionally, as stated above, we considered that both the histological and endocrinological significance of limb and body width were different; therefore, we chose linear analysis instead of volumetric analysis. Fifth, since there were various reports of investigations of associations between adrenal size and depression as mentioned above, we should have excluded patients with depression. However, the present study was retrospective and we could not diagnose depression in patients precisely from medical records. Therefore, we dealt with this problem by including “having depressive symptoms” in the questionnaire in multivariate models. Although the relationship between OSA and mood disorders was inconclusive [50,51], a greater number of participants with anxiety were in the none/mild OSA than severe OSA groupings in our cohort. The result was consistent with a previous hospital-based study with a large sample size [52]. Since significant associations between anxiety and adrenal size were not found in univariate analyses (Table 2), we did not include anxiety in the multivariate models.

In summary, we investigated the relationship between adrenal size measured by abdominal CT and sleep parameters in OSA patients. We found that the arousal index but not hypoxic parameters was independently associated with increased adrenal limb width. Our results provided evidence linking OSA and HPA axis activation in terms of a morphological change in the adrenal gland where chronic sleep fragmentation might act as a stronger stressor that alters adrenal size.

## Supporting information

S1 Table Multivariate regression analysis for mean of limbs. n = 252
Adjusted R^2^ was calculated from R^2^ after adjusting for the number of covariates in models.
Definition of abbreviations: β = standardized partial regression coefficient; MetS = metabolic syndrome; AHI = apnea-hypopnea index; ODI = oxygen desaturation index.
(DOCX)

S1 Dataset (XLSX)
