# Supplementary material for: Adrenal gland size in obstructive sleep apnea: Morphological assessment of hypothalamic pituitary adrenal axis activity
Source: PLoS One. 2019 Sep 20;14(9):e0222592. doi: 10.1371/journal.pone.0222592 (PMC6754148; doi:10.1371/journal.pone.0222592)
Supplement: S1 Table — n = 252 Adjusted R2 was calculated from R2 after adjusting for the number of covariates in models. Definition of abbreviations: β = standardized partial regression coefficient; MetS = metabolic syndrome; AHI = apnea-hypopnea index; ODI = oxygen desaturation index. (DOCX) [file pone.0222592.s001.docx]

**S1 Table. Multivariate regression analysis for mean of limbs. n = 252**

| **Mean limb width** | | | | | | | | |
| --- | --- | --- | --- | --- | --- | --- | --- | --- |
| **Variables** | **Model 1** | |  | **Model 2** | |  | **Model 3** | |
|  | **ß** | **P value** |  | **ß** | **P value** |  | **ß** | **P value** |
| Age (years) | 0.22 | <0.001 |  | 0.22 | <0.001 |  | 0.16 | <0.001 |
| Sex (men) | 0.14 | 0.024 |  | 0.14 | 0.02 |  | 0.10 | 0.09 |
| Visceral fat area (cm^2^) | 0.21 | 0.010 |  | 0.21 | 0.008 |  | 0.19 | 0.014 |
| Current smoker | 0.12 | 0.043 |  | 0.12 | 0.045 |  | 0.09 | 0.12 |
| Obesity (+) | 0.06 | 0.43 |  | 0.05 | 0.48 |  | 0.06 | 0.42 |
| MetS (+) | -0.04 | 0.53 |  | -0.04 | 0.56 |  | -0.02 | 0.80 |
| Depression (+) | 0.05 | 0.37 |  | 0.04 | 0.44 |  | 0.08 | 0.18 |
| AHI (events/h) | 0.16 | 0.019 |  | - | - |  | - | - |
| 3% ODI (events/h) | - | - |  | 0.04 | 0.63 |  | - | - |
| Time of SpO_2_ <90% (%) | - | - |  | 0.13 | 0.12 |  | - | - |
| Arousal index (events/h) | - | - |  | - | - |  | 0.29 | <0.001 |
| ***R^2^* (adjusted *R^2^*), %** | **19.0 (16.3)** | |  | **19.3 (16.3)** | |  | **23.7 (21.2)** | |

Adjusted R^2^ was calculated from R^2^ after adjusting for the number of covariates in models.

Definition of abbreviations: β = standardized partial regression coefficient; MetS = metabolic syndrome; AHI = apnea-hypopnea index; ODI = oxygen desaturation index.
